# Water and Ethanol Droplet Wetting Transition during Evaporation on Omniphobic Surfaces

**DOI:** 10.1038/srep17110

**Published:** 2015-11-25

**Authors:** Xuemei Chen, Justin A. Weibel, Suresh V. Garimella

**Affiliations:** 1 School of Mechanical Engineering and Birck Nanotechnology Center, Purdue University, West Lafayette, Indiana, 47907-2088 USA

## Abstract

Omniphobic surfaces with reentrant microstructures have been investigated for a range of applications, but the evaporation of high- and low-surface-tension liquid droplets placed on such surfaces has not been rigorously studied. In this work, we develop a technique to fabricate omniphobic surfaces on copper substrates to allow for a systematic examination of the effects of surface topography on the evaporation dynamics of water and ethanol droplets. Compared to a water droplet, the ethanol droplet not only evaporates faster, but also inhibits Cassie-to-Wenzel wetting transitions on surfaces with certain geometries. We use an interfacial energy-based description of the system, including the transition energy barrier and triple line energy, to explain the underlying transition mechanism and behaviour observed. Suppression of the wetting transition during evaporation of droplets provides an important metric for evaluating the robustness of omniphobic surfaces requiring such functionality.

Superhydrophobic surfaces encountered in nature have inspired numerous theoretical and experimental investigations of wetting behaviour on rough surfaces, leading to a variety of engineered surfaces for self-cleaning^1^, drug reduction^2^, water harvesting^3^, anti-icing^4^, and condensation heat transfer enhancement^5^,^6^,^7^,^8^. However, most micro/nano-structured surfaces designed to yield superhydrophobicity^9^,^10^,^11^,^12^,^13^ are not suitable to support non-wetting states for low surface tension liquids, such as oils and alcohols. To overcome this limitation, researchers have engineered surfaces with topographic features having specialized reentrant geometries, such as inverse trapezoidal^14^, serif-T^15^,^16^, mushroom^17^,^18^,^19^, micro-hoodoo^20^,^21^, and micro-nail^22^ structures. On such surfaces, deposited droplets remain pinned at the sharp edge of the reentrant structures, where the meniscus generates an upward force that resists droplet collapse into the surface cavities, even for low surface tension liquids. Surfaces that are capable of supporting non-wetting interfaces for both high and low surface tension liquid droplets are considered to be omniphobic.

On textured superhydrophobic or omniphobic surfaces, there are two possible wetting states: in the Cassie state^23^ a sessile liquid droplet sits on top of the surface roughness, with air pockets trapped underneath, forming a composite solid-liquid-vapour interface; in the Wenzel state^24^ the droplet penetrates into the roughness elements, fully wetting the surface. Maintaining the Cassie state and avoiding intrusion of liquid into surface asperities is essential to realizing the relevant functionality of superhydrophobic/omniphobic surfaces. However, on surfaces that support a metastable Cassie state for sessile droplets, the droplets can spontaneously transition into the Wenzel state^25^; alternatively, transition can be induced by external stimuli such as pressure^26^, vibration^27^, an electric field^28^, droplet impact^29^, or droplet evaporation^30^,^31^,^32^. Among these possible transition mechanisms, the process of droplet evaporation on superhydrophobic surfaces has garnered interest in recent years due to promising technological applications, such as harnessing droplet evaporation to improve the sensitivity of biosensors for biomedical applications^33^,^34^,^35^,^36^. Because superhydrophobic surfaces exhibit non-wetting (high contact angle) and minimum adhesion (low contact angle hysteresis) behaviour, they can be used as substrates on which to concentrate target molecules in very dilute suspensions in droplets via evaporation onto localized sensing areas, resulting in extremely high detection sensitivity. If transducers for biosensing are located on the tops of pillars on a rough surface, maintaining the Cassie state until the end of droplet evaporation – when the molecules in the droplet become significantly more concentrated – can promote interaction between the molecules and the transducers^33^,^34^,^35^,^36^. Thus, understanding the mechanism of Cassie-to-Wenzel transition en route to complete evaporation is of primary importance for the design and fabrication of functional surfaces that can sustain a stable Cassie state. Two general approaches have been proposed to explain the transition mechanism, either based on the Laplace-Capillary pressure balance^37^,^38^ or on a comparison between the interfacial energies for Cassie- and Wenzel-state droplets^30^,^39^,^40^. However, almost all studies of droplet evaporation on non-wetting surfaces have been conducted for water droplets^30^,^31^,^32^,^37^,^38^,^39^,^40^,^41^,^42^,^43^; a clear, fundamental understanding of the effect of surface roughness on the wetting transition is lacking for low surface tension organic liquids evaporating on omniphobic surfaces.

In this work, we develop an approach to fabricate omniphobic surfaces with reentrant mushroom structures on copper substrates, and systematically investigate the effects of surface topography on water and ethanol droplet evaporation. We demonstrate that by controlling the surface topography, the ethanol droplets can be preserved in a Cassie state (without transitioning into the Wenzel state) throughout their evaporation lifetime. An interfacial energy-based analysis was employed to predict the Cassie-to-Wenzel wetting transition observed in the experiments.

## Results

**Figure 1:**
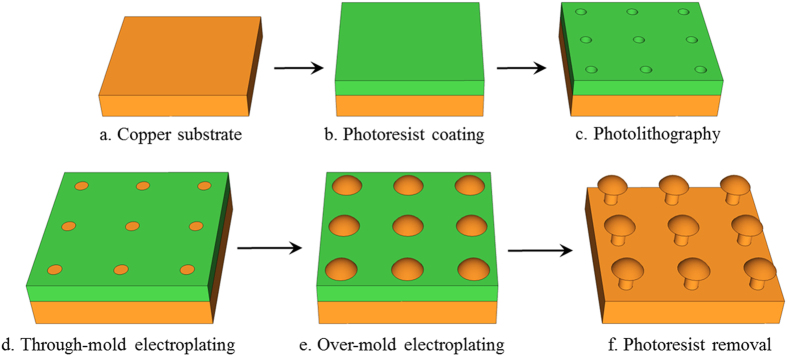
Schematic illustration of the steps in the fabrication procedure for the mushroom-structured copper surfaces. (**a**) clean copper substrate; (**b**) spin-coating of HMDS and AZ 9260 photoresist, followed by soft baking; (**c**) photoresist exposure and development; (**d**) through-mold copper electroplating; (**e**) over-mold copper electroplating; and **(f**) photoresist stripping with acetone.

**Figure 2:**
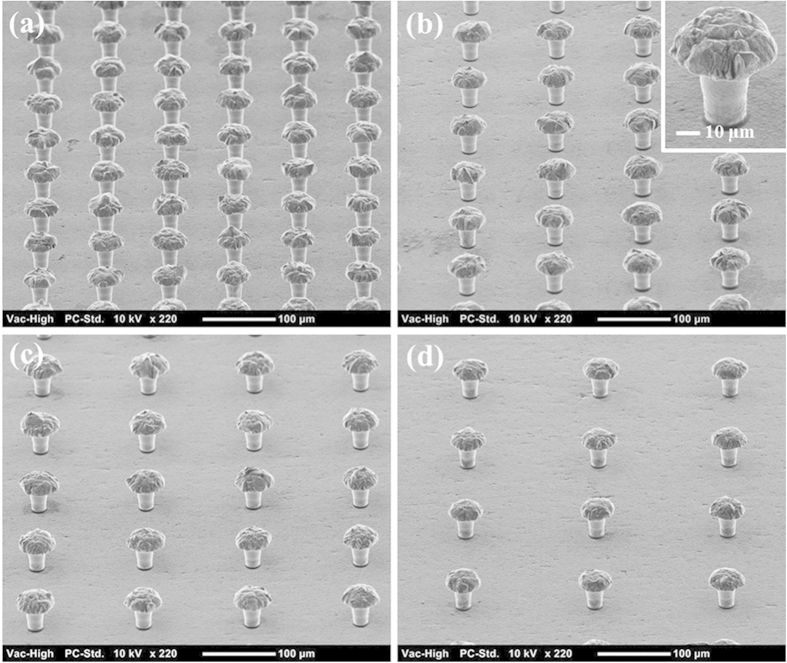
SEM images of the mushroom-structured copper surfaces. (**a**) OM-90, **(b)** OM-120, **(c)** OM-150, and **(d)** OM-180. The inset shows a close-up SEM image of a single mushroom structure.

The mushroom-structured omniphobic surfaces used in this study were fabricated on copper substrates via photolithography and electroplating processes (Fig. 1) **Methods** Section. Briefly, photolithography was used to form a 35 μm-thick photoresist mold with a square array of circular pores, and over-mold electroplating with copper was used to fill the pores and form hemispherical mounds atop the mold layer at each pore location. Copper surfaces with mushroom center-to-center spacing of 90, 120, 150, and 180 μm were fabricated by varying the photolithography mask patterns. Figure 2 shows scanning electron microscope (SEM) images of the mushroom-structured copper surfaces. These omniphobic surfaces are denoted as OM-90, OM-120, OM-150, and OM-180, based on the characteristic mushroom center-to-center spacing (pitch, *P*). For all the surfaces, the mushroom cap diameter (*D*) and height (*h*) are ~48 μm and ~15 μm; the mushroom stem diameter (*d*) and height (*H*) are ~18 μm and ~35 μm.

To assess the robustness of the Cassie state on the reentrant surfaces, a dimensionless parameter A^*^ was previously developed to quantify the resistance of the surface to the Cassie-to-Wenzel transition^20^,^21^,^22^. This dimensionless robustness parameter is the ratio of breakthrough pressure (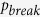, the pressure required to cause the liquid droplet to transition from the Cassie to the Wenzel state) to a reference pressure (
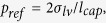
 where 
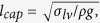
 is the capillary length of the liquid, 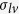 is the liquid-vapour surface tension, *ρ* is the liquid density, and *g* is the gravitational acceleration). For upright mushroom-structured surfaces, the robustness parameter A^*^ is evaluated as (Supplementary Section 3)^20^,^21^,^22^:


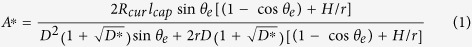


in which 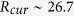
μm is the curvature radius of the mushroom cap, *θ*_*e*_ is the equilibrium contact angle on the 1H,1H,2H,2H-perfluorodecyltrichlorosilane (PFDS)-coated flat copper surface, and 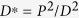 is the dimensionless spacing ratio of the structures. To theoretically achieve a Cassie state, the parameter *A*^∗^ should be larger than unity; larger *A*^∗^ values predict greater robustness.

**Figure 3:**
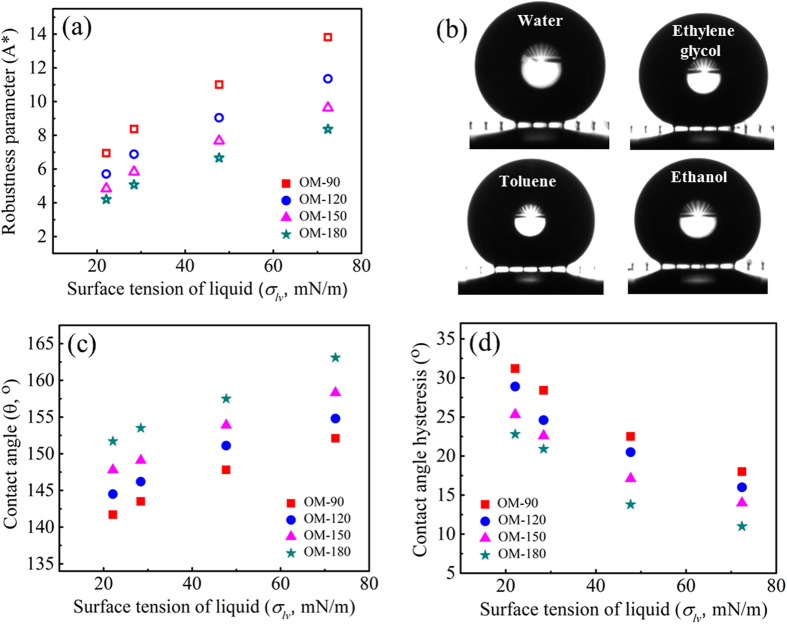
Characterization of omniphobicity on mushroom-structured surfaces. (**a**) calculated robustness parameter on each surface as a function of surface tension; **(b)** contact angle images for water, ethylene glycol, toluene, and ethanol on the OM-180 surface; and measured **(c)** contact angles and **(d)** contact angle hysteresis.

For the surfaces fabricated and shown in Fig. 2, the magnitude of *A*^∗^ was calculated for liquid droplets with different surface tensions, including water (~72.4 mN/m), ethylene glycol (~47.7 mN/m), toluene (~28.7 mN/m), and ethanol (~22.1 mN/m), and is plotted in Fig. 3a. The contact angles *θ*_*e*_ of these four liquids on PFDS-coated flat surfaces were measured to be ~115°, ~102°, ~65°, and ~54°, respectively. The robustness parameter A^*^ for all of the surfaces is larger than 1, indicating that the surfaces should be able to maintain droplets in the non-wetting Cassie state for all four liquids; the robustness increases with increasing liquid surface tension as well as decreasing mushroom center-to-center spacing. This theoretical assessment is supported by the wettability experiments. Figure 3b shows images of liquid droplets with each different surface tension on the OM-180 surface. All the droplets remain in the Cassie state, as indicated by the backlight visible through the mushroom gaps. The measured contact angle (CA) values for liquid droplets on the mushroom-structured surfaces are given in Fig. 3c. The surfaces indeed exhibit omniphobic behaviour and the CA increases with increases in the mushroom spacing as well as liquid surface tension. The OM-180 surface, on which the CA is greater than 150° over a broad range of liquid surface tensions, is considered superomniphobic. The theoretical CA was calculated for each liquid and surface using the Cassie equation (Supplementary Fig. S1 and S2). The theoretical values match the experimental observations for the tested liquids when the mushroom spacing is larger than 120 μm, whereas there is a large discrepancy between theoretical and experimental values for the larger surface tension liquids (water and ethylene glycol) when the spacing is less than 120 μm, and for the lower surface tension liquids (toluene and ethanol) when the mushroom spacing is less than 150 μm. The inconsistency between theoretical and experimental CA values may be due to the assumption in the Cassie equation that the droplet takes the shape of a spherical cap, and the contact area between the droplet and solid surface is flat^23^. In this work, the lower surface tension fluids tested may cause the droplet shape to become flattened when deposited on the surfaces (Fig. 3b); the local shape of the solid-liquid interface for the low surface tension liquids also becomes distorted on the reentrant surfaces^44^; which may also account for the inaccuracy of the Cassie equation. Figure 3d shows the measured CAH values (advancing and receding contact angles are provided in Supplementary Fig. S3). We observe that the contact angle hysteresis increases with decreasing liquid surface tension and mushroom spacing. This may be attributed to a comparatively larger pinning force for the wetting liquids and dense mushroom structures, which is proportional to the contact angle hysteresis^45^,^46^.

**Figure 4:**
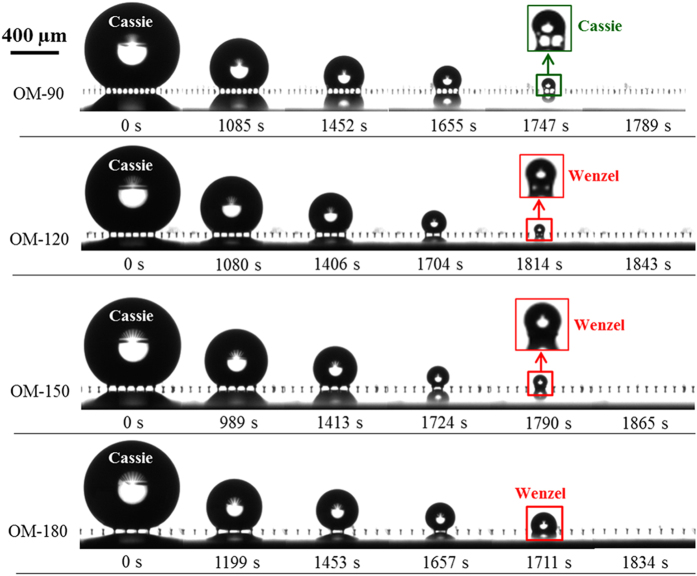
Images of water droplets evaporating on the omniphobic surfaces. On surface OM-90, the droplet stayed in the Cassie state for its entire lifetime (as indicated by the visible backlight between the mushroom structures in the magnified inset image). The droplets on surfaces OM-120, OM-150, and OM-180 undergo the Cassie-to-Wenzel transition towards the end of the evaporation period.

**Figure 5:**
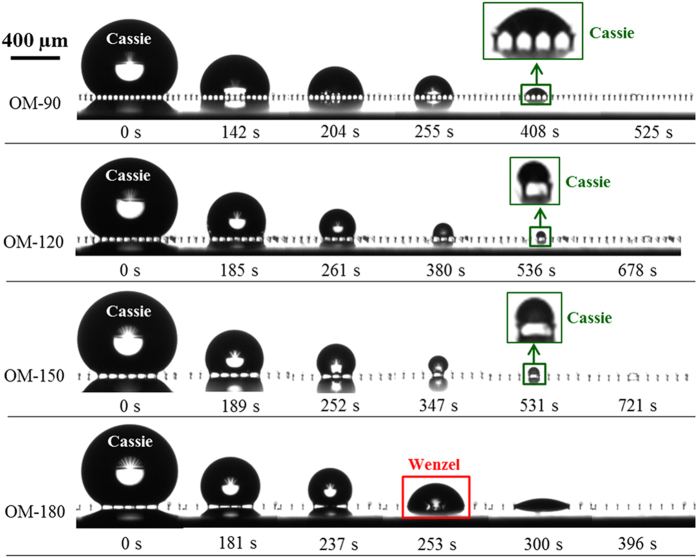
Images of ethanol droplets evaporating on the omniphobic surfaces. On surfaces OM-90, OM-120 and OM-150, no Cassie-to-Wenzel transition was observed, as shown in the magnified inset image. On surface OM-180, however, the droplet first sits in the Cassie state, and then transitions into the Wenzel state at ~253 s.

In order to study whether the mushroom-structured surfaces can preserve long-term omniphobicity, we studied the evaporation dynamics of droplets of a high surface tension liquid (water), as well as a volatile low surface tension organic liquid (ethanol), on each surface. Figure 4 shows photographs at selected times of evaporating water droplets on the four surfaces. On surface OM-90, the water droplets remain in the Cassie state throughout evaporation. There was no Cassie-to-Wenzel transition even for very small droplet sizes at the late stage of evaporation. The magnified inset image shows the droplet just prior to complete evaporation, and the backlight is visible between the mushroom structures for surface OM-90. However, the wetting transition did occur at the end of water droplet evaporation on all surfaces with a mushroom center-to-center spacing of 120 μm or larger. Figure 5 shows a similar series of photographs during the evaporation period for ethanol droplets on the same surfaces. On surfaces OM-90, OM-120, and OM-150, Cassie-to-Wenzel transition was not observed, just as for the water droplet evaporating on surface OM-90. On surface OM-180 with the largest mushroom spacing, the droplet transitioned into the Wenzel state at 253 s. Side-by-side video comparisons of water and ethanol droplets evaporating on each surface are shown in the Supplementary Movies S1–S4.

**Figure 6:**
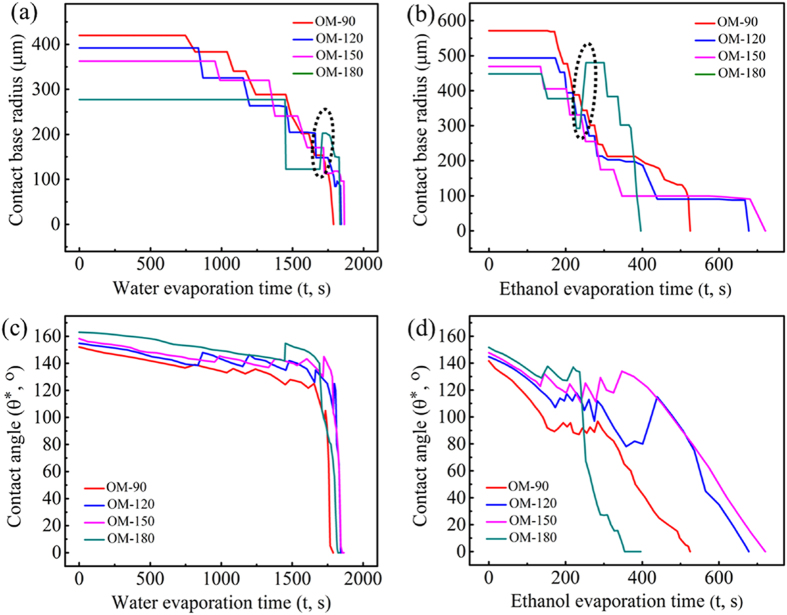
Contact base radius and contact angles for (a,c) water and (b,d) ethanol droplets, respectively, evaporating on the omniphobic surfaces as a function of time.

The temporal variations in contact base radius and contact angle for evaporating water and ethanol droplets are plotted in Fig. 6. The average evaporation lifetime for an ethanol droplet is approximately one-third that for a water droplet. For both water and ethanol droplets evaporating on the omniphobic surfaces, the contact line was initially pinned to the solid surfaces (Fig. 6a,b), leading to a decreasing CA with loss of droplet volume (Fig. 6c,d). Once the CA was reduced to some critical value (approximately the receding CA) due to evaporation, the contact line started to retract rapidly and resulted in a small sudden increase in the CA. This was followed by successive pinning and depinning of the contact line in small stepwise increments, which is referred to as “stick-slip” phenomenon^47^. The behaviour at the late stages of evaporation is a function of the surface geometry and whether or not a Cassie-to-Wenzel transition occurs. A sudden increase in the base radius for water and ethanol droplets on surface OM-180 (highlighted by the dotted ovals in Fig. 6a,b) are indicative of the Cassie-to-Wenzel transition. While a Cassie-to-Wenzel transition was observed for water droplets on surfaces OM-120 and OM-150 an abrupt change in the base radius is not noticeable due to the small size of the droplet at transition (base radius on the order of the mushroom center-to-center spacing).

## Discussion

Regarding prediction of the Cassie-to-Wenzel wetting state transition on reentrant superomniphobic surfaces, Tuteja *et al.*^21^ proposed a transition mechanism by considering the local Laplace pressure which drives the transition, and the breakthrough pressure which resists the transition. As the droplet shrinks during evaporation, the Laplace pressure of the droplet builds up. When the Laplace pressure exceeds the breakthrough pressure, the droplet sinks to a Wenzel state. Based on this proposed mechanism, we calculated the breakthrough pressure for water and ethanol droplets on each of the fabricated surfaces (Supplementary Fig. S4), as well as the Laplace pressure of the evaporating droplets as a function of time (Supplementary Fig. S5). As is shown in Fig. S5, the predictions of Cassie-to-Wenzel transition based on Tuteja *et al.*^21^ are either contrary to our experimental observations, or the predicted moments of transition do not match with the experimentally observed transition times. Therefore, the Cassie-to-Wenzel transitions do not appear to be fully described by the Laplace pressure versus breakthrough pressure balance mechanism.

**Figure 7:**
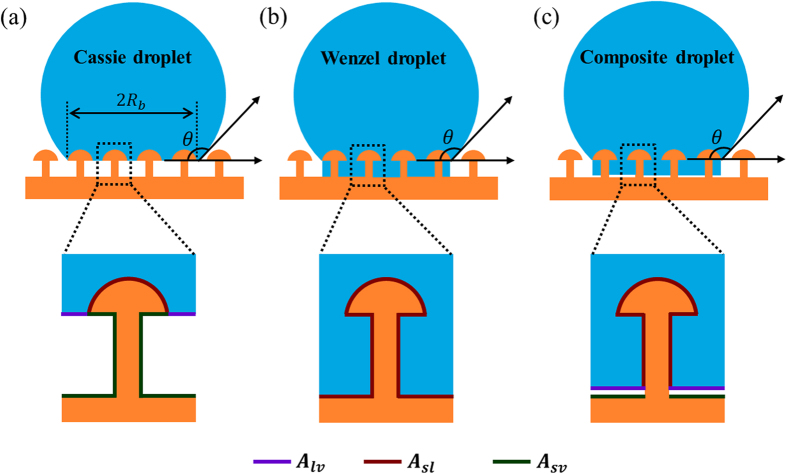
Schematic drawing of (a) Cassie-, (b) Wenzel-, and (c) composite-state droplets sitting on the mushroom-structured omniphobic surfaces. The enlarged views show the interfacial liquid-vapour, solid-liquid, and solid-vapour areas of the droplet contacting a single mushroom structure.

We then employed an alternative energy analysis method that compares the interfacial energies of droplets in both the Cassie (*E*_*C*_) and Wenzel (*E*_*W*_) states to predict the Cassie-to-Wenzel wetting transition (Fig. 7)^30^,^39^,^40^. It has been shown that the three-phase contact line for a droplet in contact with the mushroom structures is locally deformed (Supplementary Fig. S6). Under this condition, the excess energy at the triple junction (line energy) is not negligible in the energy analysis, even for large droplets^48^. Moreover, at late stages of evaporation, the droplet size is comparable to the reentrant structure, and the line energy contributes significantly to the total energy of the system^30^. Thus, a general expression for the interfacial energy of a droplet on the mushroom-structured surface is given by:


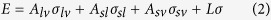


where *A* is the interfacial area associated with the liquid-vapour (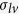), solid-liquid (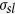), and solid-vapor (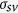) interfaces, *L* is the triple-line length, and *σ* is the line tension.

The energies of Cassie (Fig. 7a) and Wenzel (Fig. 7b) state droplets are respectively described by:


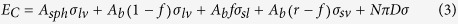



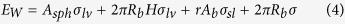


Where 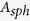 is the surface area of the spherical-cap droplet in contact with the vapour, which is expressed as 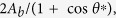 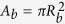 is the droplet base area, θ^*^ is the measured apparent contact angle of the evaporating droplet,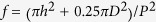 is the solid fraction of the surface (if the droplet is fully in contact with the mushroom caps and the liquid-air interface underneath the droplet is relatively flat; a discussion of these assumptions is detailed in Supplementary Section 4), 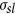 is estimated using the modified Young’s equation: 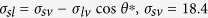 mN/m (Supplementary Fig. S7), 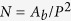 is the number of mushrooms underneath the droplet, and 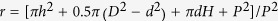 is the surface roughness.

For an evaporating Cassie-state droplet, an energy barrier must be overcome in order to transition from the Cassie to the Wenzel state. It has been supposed that this energy barrier corresponds to the energy variation between the Cassie state (*E*_C_) and a hypothetical composite state (*E*_*comp*_) where the penetrating liquid droplet almost completely fills the surface asperities but does not touch the bottom surface, and the apparent contact angle of the droplet does not change during the penetration process (Fig. 7c)^49^. The energy of a composite state droplet is given as follows:


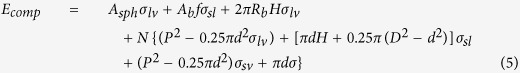


According to equations (3) and (5), the magnitude of the energy barrier separating the Cassie and the Wenzel states is calculated as:


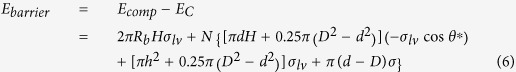


During the evaporation process, the Cassie-to-Wenzel wetting transition will occur once the energy difference 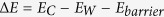 becomes equal or larger than zero. Therefore, based on the droplet base radius (*R*_*b*_) and droplet contact angles (θ^*^) shown in Fig. 6, we calculated *E*_*C*_ and *E*_*W*_ for the droplets at each moment in the evaporation process, as well as the energy barrier 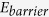 associated with the wetting transition. Note that there is no consensus in the literature for the magnitude or sign of the line tension *σ* shown in equations (3, 4, 5, 6). It has been reported that a negative line tension could stabilize the Cassie state for a droplet sitting on the reentrant surface^50^. In the current study, a value of −7 × 10^−6^ N is chosen for *σ*, which falls within the wide range (−10^−6^ N to −10^−12^ N) found in the literature^51^,^52^,^53^,^54^, and provides the best agreement with our experimental results.

**Figure 8:**
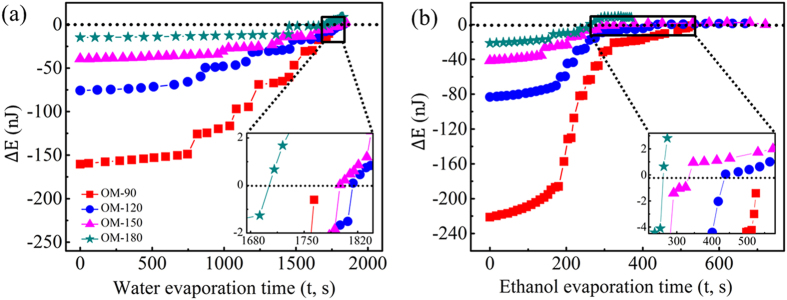
The energy differences 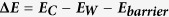 for the evaporating (a) water and (b) ethanol droplets as a function of time. The inset shows an enlarged view of energy differences at the late stages of evaporation.

The energy difference 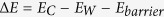 is plotted for the evaporating water and ethanol droplets as a function of time in Fig. 8. The inset shows an enlarged view of energy differences at the late stages of evaporation. For the water droplets evaporating on surface OM-90 (Fig. 8a), the energy difference Δ*E* remains negative throughout the droplet lifetimes. This indicates that the Cassie state cannot overcome the energy barrier to transition into the Wenzel state, which is in agreement with the experimental observation. On surfaces OM-120, OM-150, and OM-180, the energy differences Δ*E* are negative initially and gradually increase as the evaporation proceeds; the difference rises above zero at *t* = 1814 s, 1790 s, and 1711 s, respectively, for the three surfaces. These times correspond to the moments of Cassie-to-Wenzel transition in the experiments. These results demonstrate that the energy-based analysis presented, which takes into account the line energy and considers the transition energy barrier, can accurately predict the Cassie-to-Wenzel wetting transition for water droplets on the omniphobic surfaces. For an ethanol droplet (Fig. 8b), the energy difference Δ*E* on surface OM-90 also remains negative throughout the evaporation process, suggesting that no Cassie-to-Wenzel transition should take place, which is consistent with the experimental result. On surface OM-180, the energy difference Δ*E* is negative from 0 s to 237 s, and becomes larger than zero at 253 s. This time matches the experimentally observed Cassie-to-Wenzel wetting transition. However, the inset plot of Fig. 8b shows that on surfaces OM-120 and OM-150, the energy difference rises above the zero threshold respectively at ~439 s and ~347 s, indicating that wetting transitions should occur at these two time instants on the corresponding surfaces; this is in contradiction with our experimental observations. Since ethanol has a lower surface tension compared to water, the wetting transition tendency of ethanol droplets on surfaces OM-120 and OM-150 should exceed that of the water droplets. This counter-intuitive trend displayed in the experiments with the ethanol droplets might be explained by the ethanol residue depositing on the mushroom caps towards the end of evaporation (as demonstrated by the SEM images of these surfaces after droplet evaporation in Supplementary Fig. S8). The ethanol residue on surfaces OM-120 and OM-150 indicate that ethanol molecules are concentrated on the tops of surfaces as evaporation proceeds, which would result in the droplet pinning strongly to the solid surface. This pinning behaviour may be hindering the expected Cassie-to-Wenzel transition on surfaces OM-120 and OM-150.

To confirm that the energy analysis predicts the transition behavior on the omniphobic surfaces for a larger sampling of fluids, we conduct droplet evaporation experiments with additional low-surface-tension organic liquids, *viz.*, methanol, toluene, and heptane (Supplementary Fig. S9–S11). These results show that the interfacial energy analysis successfully predicts the wetting transition behaviour observed in the experiments. The suppression of Cassie-to-Wenzel wetting transition of low surface tension organic liquids during evaporation on the mushroom-structured omniphobic surfaces offers advantages for biosensing applications. First, the organic liquid droplet has a significantly shorter evaporation lifetime compared to a water droplet of the same initial volume (approximately one-third the evaporation time for ethanol relative to water). Second, the organic liquid droplet evaporates with a receding contact line, which would minimize the loss of target molecules when serving as a carrier liquid during the evaporative enrichment process. Third, the ability to sustain Cassie-state droplets during the late stages of evaporation could promote the interaction between the molecules and the transducers if the sensing structures are located on the tops of pillars on a rough surface. As an added benefit, the omniphobic surfaces possess excellent chemical stability (Supplementary Fig. S12).

In summary, we developed a technique to fabricate mushroom-structured omniphobic surfaces on copper substrates, and systematically investigated the effect of surface topography on the evaporation phenomena of water and ethanol droplets on the fabricated surfaces. We found that, compared to water droplets, the ethanol droplets not only have shorter evaporation lifetimes, but also inhibit the Cassie-to-Wenzel wetting transition for certain geometries at the late stages of evaporation. We present an energy-based approach that incorporates the interfacial energies, line energy, and transition energy barrier to analyze the mechanisms underlying the wetting transition behaviour observed in the experiments. We envision that maintaining the Cassie state until the end of droplet evaporation, as demonstrated for volatile organic liquid droplets on omniphobic surfaces, can be harnessed for applications such as biosensors that require this type of droplet behavior during evaporation.

## Methods

### Fabrication of Omniphobic Mushroom-Structured Surfaces

The mushroom-structured omniphobic surfaces used in this study were fabricated in the Birck Nanotechnology Center at Purdue University. The fabrication procedure includes photolithography and electroplating processes. The polished copper foil (0.675 mm thick, Alfa Aesar) was first thoroughly cleaned with deioinized (DI) water and dried with nitrogen. Hexamethyldisilazane was spin-coated onto the copper substrate at 3000 rpm for 20 s to enhance adhesion of photoresist. Subsequently, the photoresist AZ 9260 was spin-coated twice at 1000 rpm for 30 s. After each individual spin coating process, the sample was baked to semi-harden the photoresist. The first soft-bake was performed at 100 °C for 10 min, and the second soft-bake was carried out at 100 °C for 15 min. After soft-baking, the photoresist was exposed at a power of 10 mW/cm^2^ for 7.5 min using a Karl Suss MJB-3 mask aligner, and developed in diluted AZ 400 K (dilution ratio of 1:2 with DI water) for 3 min. A 35 μm-thick photoresist layer with a square array of circular pores was thus produced as a mold for subsequent copper deposition on the exposed substrate regions via pulse-electroplating. This electroplating setup was described previously in ref. 7. In this work, the electroplating was performed with a current density of 40 mA/cm^2^ for ~3 hr. The electroplating duration was chosen such that the pores became over-filled with deposited copper, which formed hemispherical mounds atop the photoresist mold layer at each pore location. After electroplating, the copper substrate was soaked in acetone for 2 min to dissolve the AZ 9260 photoresist mold and reveal the mushroom-shaped structures on the copper substrate. Using this procedure, copper surfaces with mushroom center-to-center spacing of 90, 120, 150, and 180 μm were fabricated by varying the mask patterns. Surface fluorination was performed by immersing the samples in hexane solution of 0.5 wt% 1H,1H,2H,2H-perfluorodecyltrichlorosilane (PFDS) for 1 hr, followed by heat treatment at ∼150 °C on a hotplate for 1 hr.

### Surface Morphology Characterization

The detailed surface morphology of each fabricated surface was characterized using a NeoScope JCM-5000 benchtop scanning electron microscope.

### Surface Wettability Characterization

Static and dynamic apparent contact angles were measured using a Ramé-Hart goniometer (Model 590). Liquids with a wide range of surface tension values, *viz.*, water (~72.4 mN/m), ethylene glycol (~47.7 mN/m), toluene (~28.7 mN/m), and ethanol (~22.1 mN/m), were used as test fluids. Droplets of ~2 μL volume were gently deposited on the samples with a pipette, and the static contact angle (CA) was measured using the goniometer optics. The contact angle hysteresis (CAH) was measured using a tilt-stage method. A ~2 μL droplet was placed on the sample, and the stage was tilted slightly until the droplet began to slide along the surface. The contact angles at the upstream and downstream droplet edges on the inclined surface at the moment when the droplet just starts to move down the surface were considered as the advancing and receding contact angles, respectively; the difference between these angles gives the CAH. To ensure repeatability of the results, all experiments were repeated five times at different locations on each sample (the errors were less than ± 2° for all cases), and the mean CA and CAH are reported.

### Droplet Evaporation

The evaporation of water and ethanol droplets on each surface was carried out at room temperature (~22 °C) at a relative humidity of ~45%. During the experiment, a ~ 2 μL droplet was gently deposited on the as-fabricated surfaces. The contact base radius and the contact angle were recorded in real-time using the goniometer.

## Additional Information

**How to cite this article**: Chen, X. *et al.* Water and Ethanol Droplet Wetting Transition during Evaporation on Omniphobic Surfaces. *Sci. Rep.* **5**, 17110; doi: 10.1038/srep17110 (2015).

## Supplementary Material

Supplementary Information

Supplementary Movie S1

Supplementary Movie S2

Supplementary Movie S3

Supplementary Movie S4
