# Supplementary material for: Water and Ethanol Droplet Wetting Transition during Evaporation on Omniphobic Surfaces
Source: Sci Rep. 2015 Nov 25;5:17110. doi: 10.1038/srep17110 (PMC4658527; doi:10.1038/srep17110)
Supplement: Supplementary Information [file srep17110-s1.pdf]

Supplementary Information

Water and Ethanol Droplet Wetting Transition during Evaporation on Omniphobic Surfaces

*Xuemei Chen, Justin A. Weibel, and Suresh V. Garimella**

School of Mechanical Engineering and Birck Nanotechnology Center, Purdue University, West Lafayette, Indiana, 47907-2088 USA

* Correspondence and requests for materials should be addressed to S.V.G (sureshg@purdue.edu)

1. Comparison of theoretical and experimental contact angles

As demonstrated in the paper, liquid droplets with surface tension values ranging from ~ 22.1 to ~ 72.4 mN/m stay in the Cassie state on our mushroom-structured surfaces (Fig. S1). Therefore, we use the Cassie equation to predict the theoretical contact angles θ_C.[1] The equation is expressed as:

$$cos\theta_C = f(cos\theta_e + 1) - 1 \qquad \text{(S1)}$$

where θ_e is the contact angle on the flat surface, and $f = (\pi h^2 + 0.25\pi D^2)/P^2$ is the solid fraction when assuming the droplet fully contacts the mushroom caps (Fig. S1b).

For ethanol, toluene, ethylene glycol, and water, the contact angles θ_e on the flat surfaces

coated with 1H,1H,2H,2H-perfluorodecyltrichlorosilane (PFDS) were measured to be ~ 54°, ~ 65°, ~ 102°, and ~ 115°, respectively. A comparison of theoretical and experimental CA as a function of mushroom spacing is provided in Fig. S2.

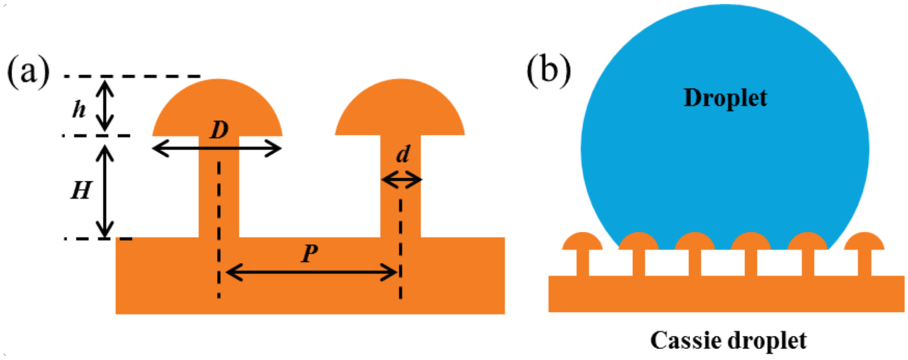

Figure S1. Schematic drawings (a) defining the characteristic geometric features of a mushroom-structured surface, and (b) showing a Cassie-state droplet sitting on the mushroom-structured surface.

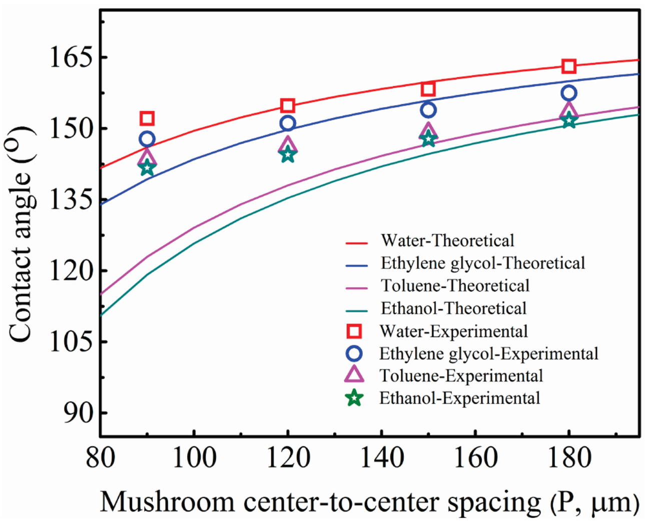

Figure S2. Comparison of theoretical and experimental contact angles for liquid droplets on surfaces OM-90, OM-120, OM-150, and OM-180.

2. Receding and advancing contact angles

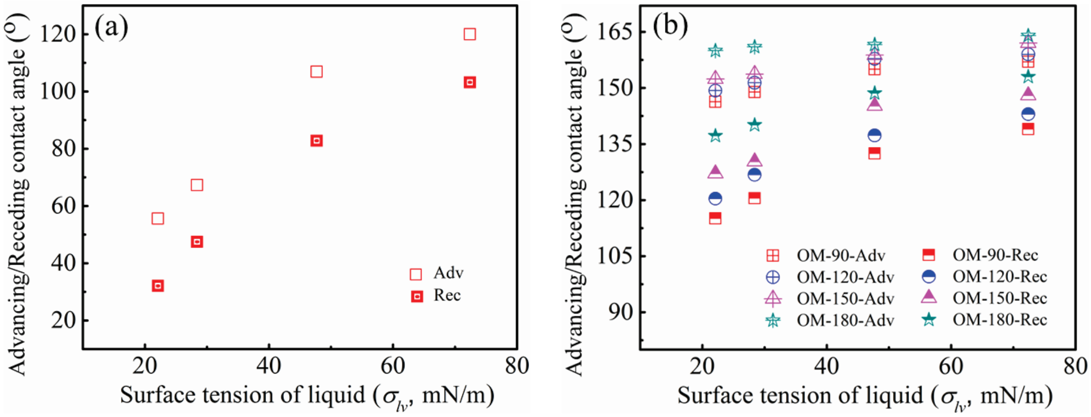

Figure S3. The measured advancing and receding angles on the (a) flat surface and (b) omniphobic surfaces for water, ethylene glycol, toluene, and ethanol liquid droplets.

3. Laplace versus breakthrough pressure prediction of Cassie-to-Wenzel transition

The Laplace-breakthrough mechanism describes the transition by considering the magnitude of Laplace pressure and the breakthrough pressure. The breakthrough pressure P_{break} (the pressure required to cause liquid droplet transition from the Cassie to the Wenzel state) is scaled against a reference pressure with a scale factor A^*:

$$P_{break} = A^* \cdot P_{ref} \tag{S2}$$

in which P_{ref} is the reference pressure ($P_{ref} = 2\sigma_{lv}/l_{cap}$, where $l_{cap} = \sqrt{\sigma_{lv}/\rho g}$ is the capillary length of the liquid, σ_{lv} is the liquid-vapor surface tension, ρ is the liquid density, and g is the gravitational acceleration). The scale factor A^* is a surface robustness parameter, which is a measure of the robustness to the Cassie-to-Wenzel transition. The robustness parameter A^* is determined by two design parameters, the robustness height (H^*) and robustness textured angle

(T^*).[2,3] For the case of upright mushroom-structured surfaces, they are respectively expressed as follows:

$$H^* = \frac{2R_{cur}l_{cap}}{D^2(1+\sqrt{D^*})}[(1-cos\theta_e)+H/R_{cur}] \tag{S3}$$

$$T^* = \frac{l_{cap}\sin\theta_e}{D(1+\sqrt{D^*})} \tag{S4}$$

$$\frac{1}{A^*} = \frac{1}{H^*} + \frac{1}{T^*} \tag{S5}$$

where R_{cur} is the curvature radius of the mushroom cap (~26.7 μm in this work), and $D^* = P^2/D^2$ is the dimensionless spacing ratio of the structures.

Combining equations (S3)-(S5), A^* is derived as:

$$A^* = \frac{2R_{cur}l_{cap}\sin\theta_e[(1-cos\theta_e)+H/r]}{D^2(1+\sqrt{D^*})si\,n\,\theta_e+2rD(1+\sqrt{D^*})[(1-cos\theta_e)+H/r]} \tag{S6}$$

According to equations (S2) and (S6), we calculated the breakthrough pressure for water and ethanol droplets on each of the fabricated surfaces (see Fig. S4).

The Laplace pressure of the droplet is given by:

$$P_{Laplace} = \frac{2\sigma_{lv}}{R_{drop}} \tag{S7}$$

where R_{drop} is the droplet radius, which is calculated based on the droplet base radius R_b and droplet contact angle θ using the relation $R_{drop} = R_b/sin\theta$. Based on the measured temporal droplet contact base radius and contact angle (Figure 6 in the primary manuscript), the calculated Laplace pressures for the evaporating water and ethanol droplets as a function of time are shown in Fig. S5. From the enlarged view of Fig. S5 a and b, we note that for all of the surfaces other than OM-180, the Laplace pressures for both water and ethanol droplets exceed the breakthrough pressure, indicating that wetting transitions should occur. This is contrary to the experimental observations for the water droplet evaporating on surface OM-90, and the ethanol droplets evaporating on surfaces OM-90, OM-120, OM-150, for which no Cassie-to-Wenzel transition

was observed. Even for surfaces OM-120, OM-150, and OM-180 on which water droplet wetting transition occurred, the moments at which the Laplace pressures exceed the breakthrough pressure (t = 1665 s, 1650 s, and 1453 s, respectively) are not in agreement with the experimentally observed Cassie-to-Wenzel wetting transitions (t = 1814 s, 1790 s, and 1711 s, respectively).

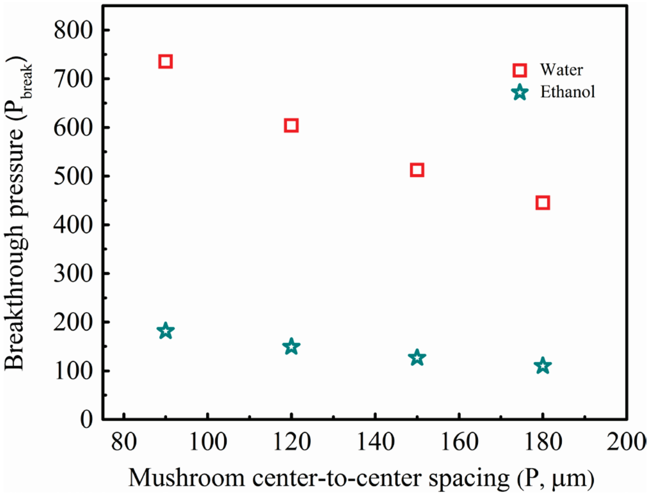

Figure S4. Breakthrough pressure for water and ethanol droplets on the mushroom-structured omniphobic surfaces.

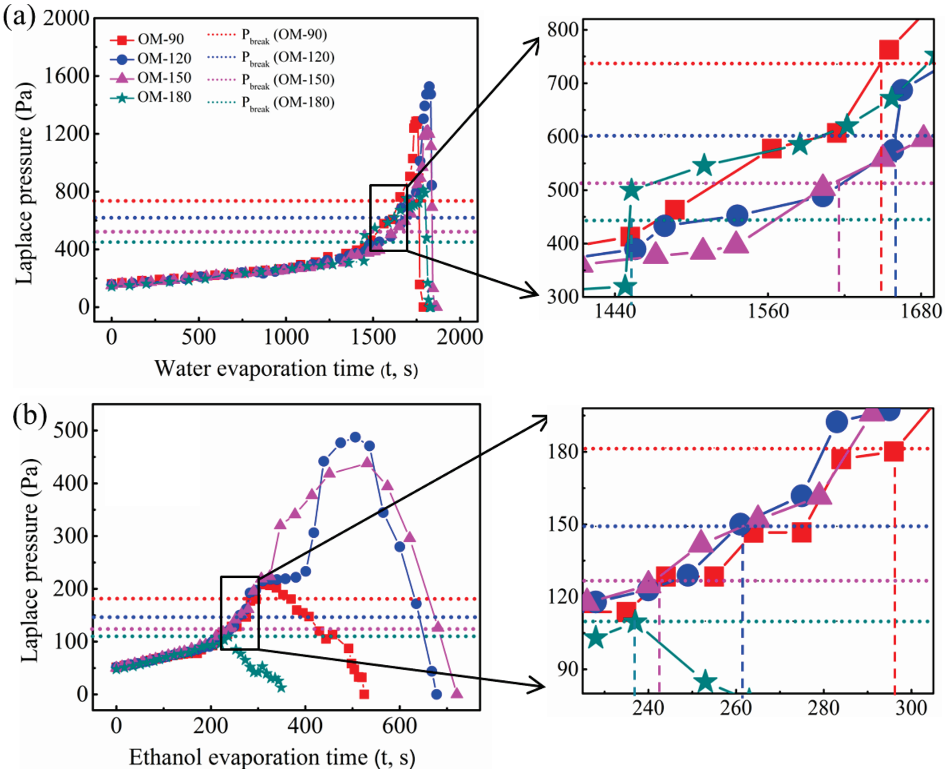

Figure S5. Calculated Laplace pressures in the evaporating (a) water and (b) ethanol droplets as a function of time. The dashed horizontal lines are the calculated breakthrough pressures for droplets on surfaces OM-90, OM-120, OM-150, and OM-180. The dashed vertical lines in the enlarged-view plots correspond to the times when the Laplace pressure exceeds the breakthrough pressure.

4. Visualization of the three-phase contact line during evaporation

We observed the three-phase contact line during the droplet evaporation process using a high-magnification lens (VH-Z100R, Keyence) that was mounted on a CCD camera (EO-5023M, Edmund Optics). Figure S6 shows side-view images of a water droplet evaporating on surface OM-90. During evaporation, the droplet contact line recedes in a stepwise fashion, jumping inward from pillar to pillar. When the droplet shrinks to a sufficiently small size, the Laplace pressure forces the droplet to penetrate into the surface asperities; the droplet remains pinned at

the edges of the reentrant pillars, generating an upward surface tension force acting along the droplet outer rim. Thus, a curved interface is observed under the droplet at t = 1427 s. Because the pillar is tall enough that this curved liquid-air interface under the droplet cannot touch the bottom substrate, the Cassie-to-Wenzel transition is ultimately suppressed (t = 1443 s). Although we can qualitatively observe slight deformation of the three-phase contact line during evaporation, we assume that the droplet fully contacts the mushroom caps (consistent with these images) and the liquid-air interface underneath the droplet is relatively flat for the purposes of predicting wetting transition.

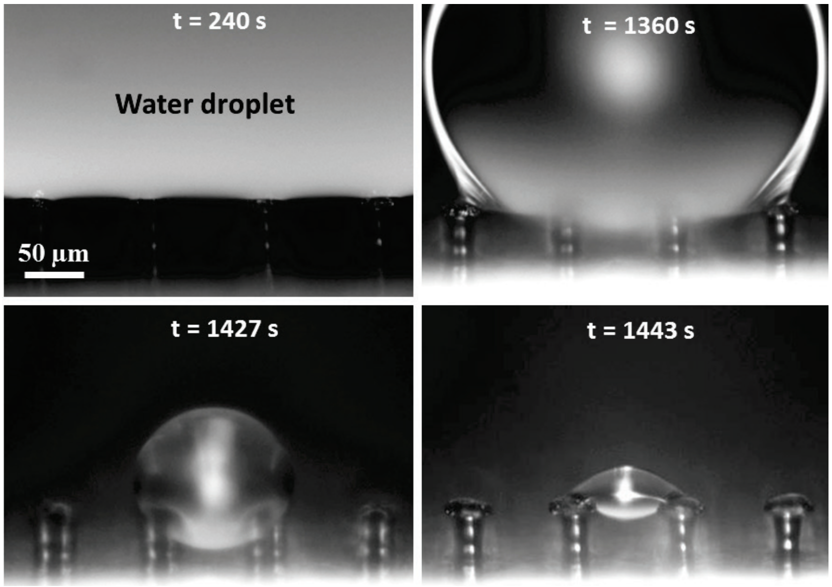

Figure S6. Images of water droplets evaporating on surface OM-90.

5. Estimation of the PFDS coating surface energy

On a chemically homogeneous and smooth surface, the wetting of a given liquid is indicated by contact angle, which is expressed by Young's equation:[4]

$$\cos\theta_e = (\sigma_{sv} - \sigma_{sl})/\sigma_{lv} \quad \text{(S8)}$$

The work required to separate the liquid and solid (work of adhesion) is described by Dupre's equation:[5]

$$W_{sl} = \sigma_{sv} + \sigma_{lv} - \sigma_{sl} \quad \text{(S9)}$$

Combining equations (S8) and (S9) yields the Young-Dupre equation:

$$W_{sl} = \sigma_{lv}(1 + cos\theta_e) \quad \text{(S10)}$$

The law of Berthelot assumes that the work of adhesion between solid and liquid is equal to the geometric mean of the cohesion work of a solid and the cohesion work of the liquid, yielding:[6,7]

$$W_{sl} = \sqrt{W_{ss}W_{ll}} = \sqrt{2\sigma_{sv} \cdot 2\sigma_{lv}} \quad \text{(S11)}$$

According to equations (S10) and (S11), we get the following expression:

$$2\sqrt{\sigma_{sv}\sigma_{lv}} = \sigma_{lv}(1 + cos\theta_e) \quad \text{(S12)}$$

Rearranging equation (12) gives:

$$(1 + cos\theta_e)^2 = (4/\sigma_{lv}) \cdot \sigma_{sv} \quad \text{(S13)}$$

Equation (S13) shows that the term $(1 + cos\theta_e)^2$ is a linear function of $4/\sigma_{lv}$, and the slope of this line is the solid surface energy σ_{sv}.[8] Using the Young contact angle θ_e and surface tension σ_{lv} of the four liquids considered in the current study (*viz.*, ethanol, toluene, ethylene glycol, and water), we plot the linear relation between $(1 + cos\theta_e)^2$ and $4/\sigma_{lv}$ in Fig. S7; the surface energy of the PFDS coating (σ_{sv}) is estimated as ~ 18.4 mN/m.

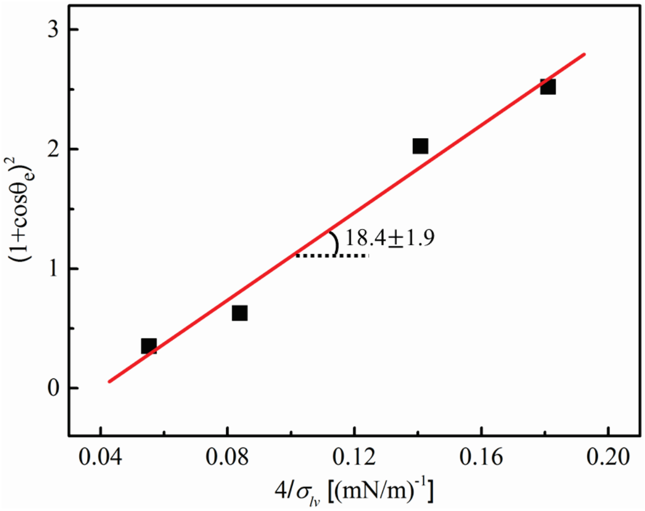

Figure S7. Estimation of the PFDS coating surface energy (slope of line).

6. SEM images of the surfaces before and after ethanol droplet evaporation

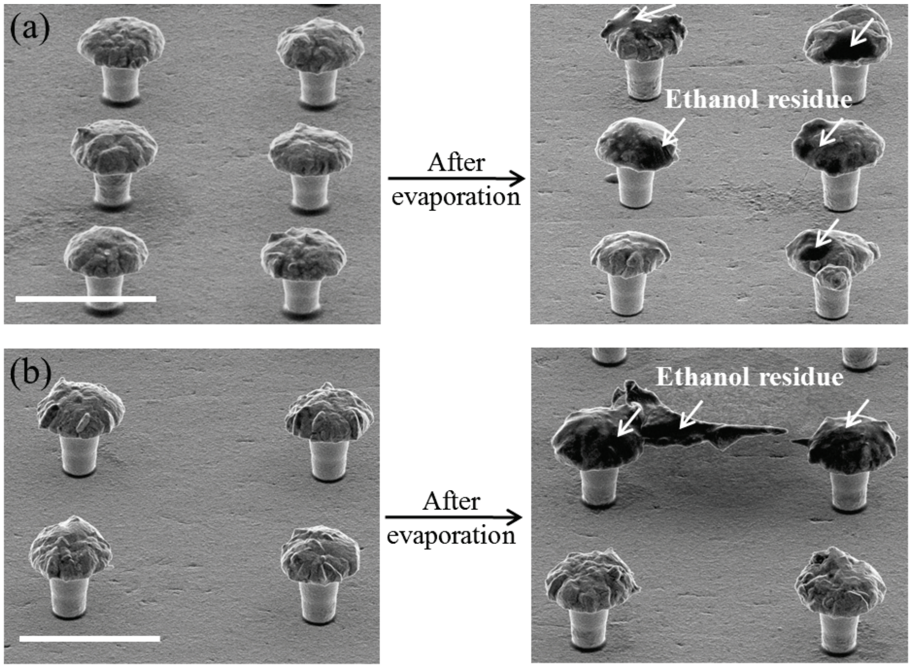

Figure S8. SEM images of surfaces (a) OM-120 and (b) OM-150 before and after ethanol droplet evaporation. The images show that the ethanol droplet deposited some residual material

(indicated by the white arrows) on the mushroom caps where the droplets resided at the end of evaporation. The scale bars are 100 μm.

7. Evaporation of other organic liquid droplets and the corresponding energy analysis

Droplets of three other volatile, low surface tension liquids, *viz.*, methanol, toluene, and heptane, are evaporated on the omniphobic surfaces (OM-90 and OM-180). Figure S9 shows photographs of the evaporating droplets on the surface OM-90 at selected times. All three droplets remain in the Cassie state throughout evaporation; Cassie-to-Wenzel transition is not observed for even very small droplet sizes at the late stages of evaporation, as indicated by the backlight visible between the mushroom structures in the magnified inset images. Figure S10 shows a similar series of photographs for evaporation of the same liquids on surface OM-180. The droplets sit in the Cassie state at first, and ultimately transition to the Wenzel state.

The interfacial energy analysis presented in the paper is used to explain the wetting transition behavior. Figure S11 shows the energy difference $\Delta E = E_c - E_w - E_{barrier}$ as a function of time for the methanol, heptane, and toluene droplets. As shown in the inset of Fig. S11a, the energy differences ΔE for all three droplets remain negative throughout the droplet lifetimes on surface OM-90, indicating that Cassie-to-Wenzel transition should not occur; this is consistent with the experimental results. On surface OM-180 (Fig. S11b), the energy differences ΔE are negative initially and gradually increase as evaporation proceeds; ΔE crosses zero at the times corresponding to the moment of Cassie-to-Wenzel transition observed experimentally.

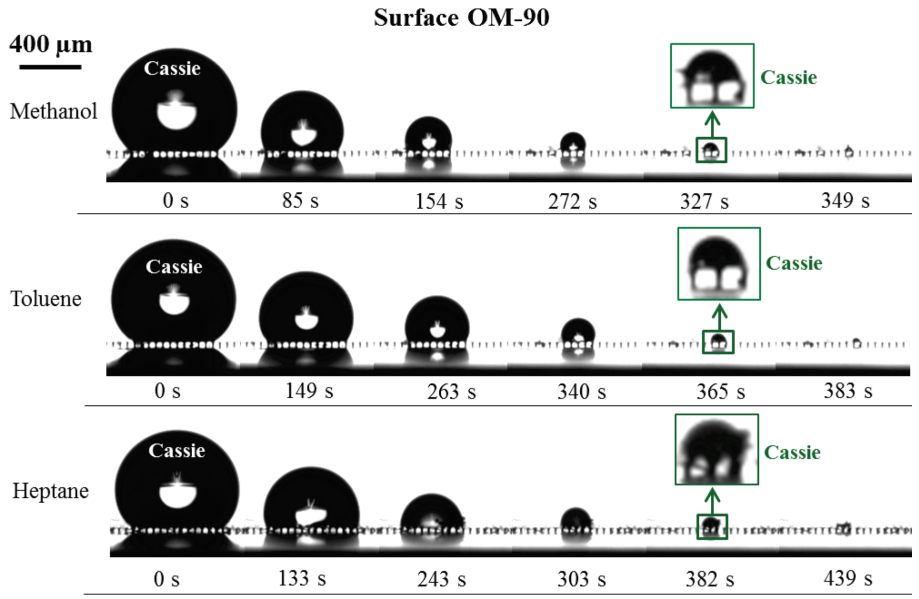

Figure S9. Images of methanol, toluene, and heptane droplets evaporating on surface OM-90. All the droplets remain in the Cassie state for their entire lifetime, as indicated by the backlight visible between the mushroom structures in the magnified inset images.

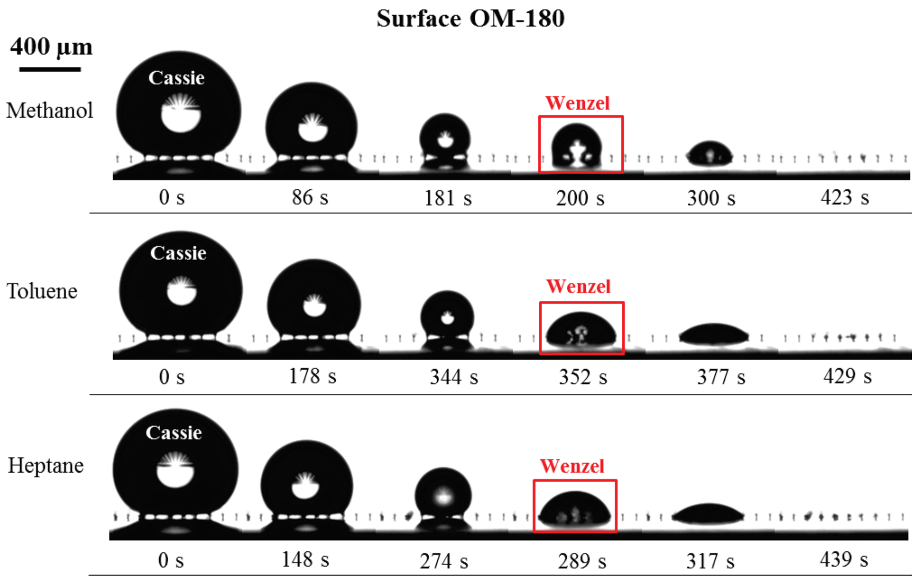

Figure S10. Images of methanol, toluene, and heptane droplets evaporating on surface OM-180. All the droplets are initially in the Cassie state and ultimately transition to the Wenzel state at ~ 200 s, 352 s, and 289 s, respectively.

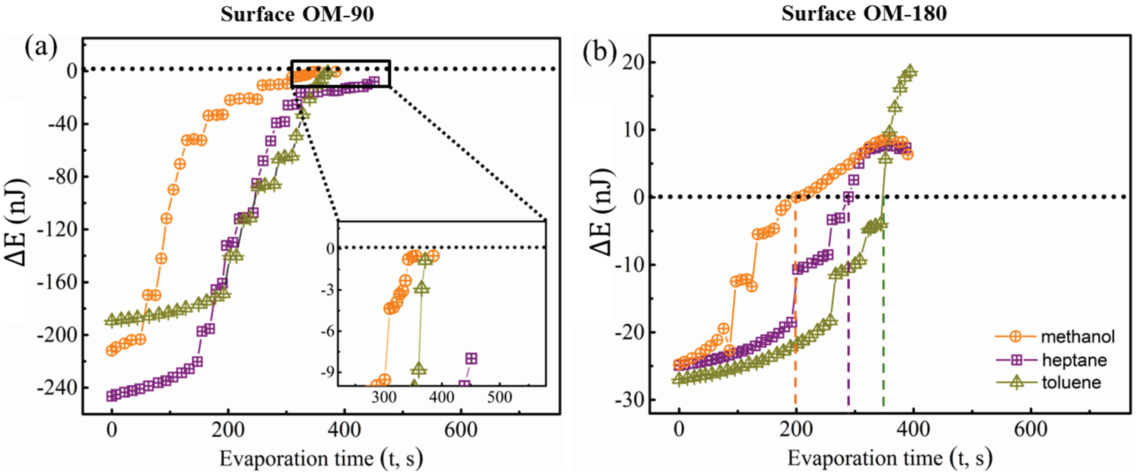

Figure S11. The energy differences *ΔE* as a function of time for methanol, heptane and toluene droplets evaporating on surfaces (a) OM-90 and (b) OM-180. The inset of the left figure shows an enlarged view of energy differences at the late stages of evaporation. The dashed vertical lines in the right figure correspond to the times at which $\Delta E = 0$.

8. Surface chemical stability test

The chemical stability of the fabricated omniphobic copper surfaces was assessed by exposing samples to different chemical environments, namely, acidic water (pH = 2) and alkaline water (pH = 12). The surfaces were immersed into these solutions and periodically removed to monitor evolution of the wetting properties through CA and CAH measurements. Figure S12 includes representative plots of the variation of CA and CAH with immersion time for surface OM-90. As shown in this figure, the contact angles of water and ethanol droplets remain almost

constant as the immersion time increases (see Fig. S12a and b); however, the CAH for both liquids slightly increases with immersion time (an increase of ~ 5-7° after 65 hr, see Fig. S12c and d). Overall, the surface maintains its omniphobicity after immersion in acidic and alkaline water for ~ 65 hr.

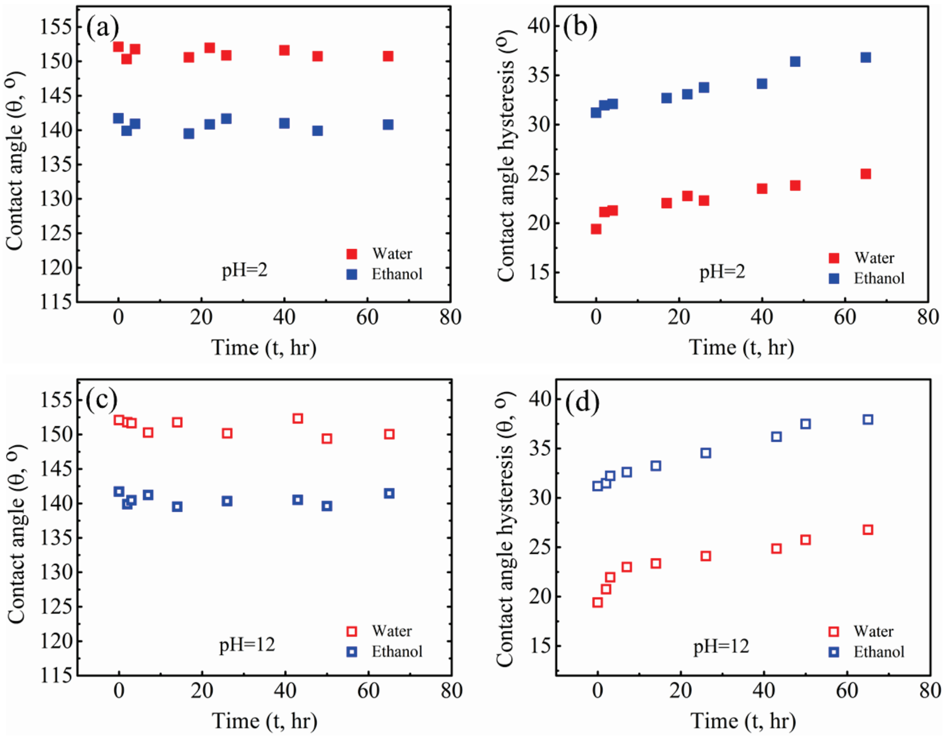

Figure S12. Contact angle and contact angle hysteresis variations for surface OM-90 in (a, b) acidic (pH = 2) and (c, d) alkaline (pH = 12) environments.

Movies:

Side-by-side video comparisons of water and ethanol droplets evaporating on surfaces OM-90, OM-120, OM-150, and OM-180 are shown in Movies S1-S4.

References

1. Cassie, A. B. D. & Baxter, S. Wettability of porous surfaces. *Trans. Faraday Soc.* **40**, 546-551 (1944).
2. Tuteja, A., Choi, W., Mabry, J. M., McKinley, G. H. & Cohen, R. E. Robust omniphobic surfaces. *Proc. Natl. Acad. Sci. U.S.A.* **105**, 18200-18205 (2008).
3. Grigoryev, A., Tokarev, I., Kornev, K. G., Luzinov, I. & Minko, S. Superomniphobic magnetic microtextures with remote wetting control. *J. Am. Chem. Soc.* **134**, 12916-12919 (2012).
4. Young, T. An essay on the cohesion of fluids. *Philos. Trans. R. Soc. Lond.* **95**, 65-87 (1805).
5. Dupré, A. & Dupré, P. *Théorie mécanique de la chaleur*. (Gauthier-Villars, 1869).
6. Berthelot, D. *Compt. Rend. (Paris)* **126**, 1857 (1898).
7. Żenkiewicz, M. Methods for the calculation of surface free energy of solids. *Int. J. Precis. Eng. Man.* **24**, 137-145 (2007).
8. Yanagisawa, K., Sakai, M., Isobe, T., Matsushita, S. & Nakajima, A. Investigation of droplet jumping on superhydrophobic coatings during dew condensation by the observation from two directions. *Appl. Surf. Sci.* **315**, 212-221 (2014).
